# The Characteristics Variation of Hepatic Progenitors after TGF-*β*1-Induced Transition and EGF-Induced Reversion

**DOI:** 10.1155/2016/6304385

**Published:** 2016-02-03

**Authors:** Ping Wang, Min Cong, Tianhui Liu, Aiting Yang, Guangyong Sun, Dong Zhang, Jian Huang, Shujie Sun, Jia Mao, Hong Ma, Jidong Jia, Hong You

**Affiliations:** ^1^Liver Research Center, Beijing Friendship Hospital, Capital Medical University, Beijing Key Laboratory of Translational Medicine in Liver Cirrhosis & National Clinical Research Center of Digestive Diseases, Beijing 100050, China; ^2^Municipal Laboratory for Liver Protection and Regulation of Regeneration, Capital Medical University, Beijing 100050, China

## Abstract

Profibrogenesis cytokine, transforming growth factor- (TGF-) *β*1, induces hepatic progenitors experiencing epithelial to mesenchymal transition (EMT) to matrix synthesis cells, even tumor initiating cells. Our previous data found that epidermal growth factor (EGF) blocks and reverses TGF-*β*1-induced transition. The aim of this study is to determine the characteristic changes of hepatic progenitors after TGF-*β*1-induced transition and EGF-induced reversion. Hepatic oval cells, rat hepatic progenitors, were isolated from rats fed a choline-deficient diet supplemented with ethionine. TGF-*β*1-containing medium was used for inducing EMT, while EGF-containing medium was used for reversing EMT. During TGF-*β*1-induced transition and EGF-induced reversion, hepatic oval cells sustained their progenitor cell marker expression, including *α*-fetoprotein, albumin, and cytokeratin-19. The proliferation ability and differentiation potential of these cells were suppressed by TGF-*β*1, while EGF resumed these capacities to the level similar to the control cells. RNA microarray analysis showed that most of the genes with significant changes after TGF-*β*1 incubation were recovered by EGF. Signal pathway analysis revealed that TGF-*β*1 impaired the pathways of cell cycle and cytochrome P450 detoxification, and EGF reverted TGF-*β*1 effects through activating MAPK and PI3K-Akt pathway. EGF reverses the characteristics impaired by TGF-*β*1 in hepatic oval cells, serving as a protective cytokine to hepatic progenitors.

## 1. Introduction

Hepatic stem cells that resided in the canal of Hering represent a small population of the cells comprising a healthy liver. Only under certain pathophysiology conditions, when the proliferation capacity of mature hepatocytes is compromised, are quiescent hepatic stem cells activated and changed into hepatic progenitors to assist in liver restoration [1, 2]. The number of hepatic progenitors is correlated with the degree of chronic hepatitis and the stage of liver fibrosis [3, 4]. Hepatic progenitors are responsive to many growth and differentiation factors [5], and the behavior of hepatic progenitors, either regenerating the liver or promoting fibrogenesis, is regulated and determined by the microenvironment in the injured liver [1, 2]. As a major fibrogenesis cytokine in the liver, transforming growth factor-*β*1 (TGF-*β*1) participates in restraining of liver regeneration and plays a dual role in liver carcinogenesis [6]. Although hepatic progenitors, also called hepatic oval cells in rat, are less sensitive to TGF-*β*1 than mature hepatocytes [7], TGF-*β*1 could cause hepatic progenitors obtaining myofibroblast cells phenotype and expressing collagen I through epithelial-mesenchymal transition* in vitro* [8]. And chronic and constant TGF-*β* exposure to rat liver progenitor-like cell line, WB-F344, makes them obtain tumor initiating properties [9]. These facts propose that it is difficult for hepatic progenitors to regenerate the liver in microenvironment of severe liver fibrosis except control and clearance of the underlying causative etiology.

As we know, epidermal growth factor (EGF) is an endogenous cell proliferation cytokine, which played a central role in regulating hepatocyte growth and liver regeneration [10]. It has been shown that mouse experimental liver fibrosis [11, 12] and hepatic stellate cell activation could be alleviated by heparin-binding EGF-like growth factor [11]. Our previous results show that EGF is a specific cytokine which could suppress TGF-*β*1-induced EMT initiation and drives mesenchymal-epithelial transition (MET) in the cells that have experienced EMT [13], revealing the plasticity of progenitor cells. Hepatic progenitors obtain the myofibroblast phenotype after EMT and lose this phenotype after MET, yet the variation of progenitor cell characteristics is still not clear during the EMT/MET process. Therefore, the aim of the present study is to determine the progenitor cell marker expression, proliferation capacity, and differentiation potential of hepatic progenitors in TGF-*β*1-induced EMT transition and EGF-induced reversion.

## 2. Materials and Methods

### 2.1. Cell Culture

Hepatic oval cells were isolated by collagenase perfusion and discontinuous gradient centrifugation from rats fed with a choline-deficient diet supplemented with ethionine for 6 weeks. Isolated cells were positive for the progenitor-specific markers OV-6, *α*-fetoprotein (AFP), and Dlk, as well as the hepatocyte markers albumin (ALB) and the cholangiocyte markers cytokeratin (CK) 19 as described previously by Wang et al. [8, 14]. Hepatic oval cells were cultured and maintained at 37°C with 5% CO_2_ in oval cell culture medium, which was composed of DMEM/Ham's F12 (Gibco) supplemented with 10% FBS (Gibco), 1 ng/mL EGF, (PeProTech), 0.5 ng/mL stem cell factor (SCF; PeProTech), and 100 U/mL penicillin and streptomycin as described previously by Wang et al. [8, 14]. For TGF-*β*1 treatment, the cells were cultured in 10% FBS-DMEM/F12 medium containing 1 ng/mL TGF-*β*1 (PeProTech) for 16 days with the medium replaced every 2 days. To examine the reversion effect of EGF, the medium was replaced with 10% FBS-DMEM/F12 medium containing 1 ng/mL EGF (PeProTech) and changed every 2 days up to 7 days.

### 2.2. RT-PCR

Total RNA extraction, reverse transcription, polymerase-chain reaction, and data analysis were carried out according to Wang et al. [14] as reported previously. The primers are listed in the supplementary materials (Supplementary Table S1 in Supplementary Material available online at http://dx.doi.org/10.1155/2016/6304385).

### 2.3. Western Blot

Whole-cell extracts were prepared using SDS lysis buffer (0.1 M Tris-HCl, 4% SDS, 0.2% bromophenol blue, and 5%  *β*-mercaptoethanol) with proteinase inhibitor cocktail (Sigma-Aldrich). Protein concentrations were determined by a Micro BCA*™* protein assay reagent kit (Pierce). After boiling for 10 min in sample buffer with sodium dodecyl sulfate and *β*-mercaptoethanol, the protein sample (7 *μ*g) was electrophoresed through 10% acrylamide gels and transferred electrophoretically to nitrocellulose membranes (Amersham Biosciences). After blocking with 5% nonfat milk in wash buffer (20 mM Tris-HCl, 100 mM NaCl, 0.05% Tween20) for 2 hrs, the membranes were incubated at 4°C overnight with primary collagen I antibody (Col-I) at 1 : 2000 (Abcam), *α*-SMA antibody at 1 : 2000 (Abcam), AFP antibody at 1 : 2000 (R&D Systems), ALB antibody at 1 : 2000 (R&D System), CK19 antibody at 1 : 2000 (R&D Systems), or *β*-actin antibody at 1 : 6000 (Sigma-Aldrich). After three washes with wash buffer, the membranes were incubated for 1 hr with corresponding horseradish peroxidase-conjugated secondary antibody at 1 : 4000 (Sigma-Aldrich). Detection and quantification of bands were performed using the Molecular Imager Chemi Doc*™* XRS+ with Image Lab Software version 3.0 (Bio-Rad).

### 2.4. Immunocytochemistry

For immunocytochemistry analysis, cells were fixed with 4% paraformaldehyde in phosphate-buffered saline (PBS) for 20 min. After permeabilization with 0.3% Triton X-100 in PBS and blocking with normal animal serum, the cells were incubated overnight at 4°C with mouse anti-AFP antibody (1 : 200; R&D Systems), mouse anti-ALB antibody (1 : 200; R&D Systems), mouse anti-CK19 antibody (1 : 200; R&D Systems), or rabbit anti-Ki-67 antibody (1 : 200; Abcam). After three washes with PBS, the primary antibodies were detected with corresponding Alexa Fluor-conjugated anti-IgG (1 : 400; Molecular Probes). The nuclei were counterstained with 4′, 6-diamidino-2-phenylindole (DAPI; Sigma-Aldrich). All cell counts were performed on blind-coded samples. We counted both the total cell numbers (at least 500) based upon DAPI-positive nuclei and the numbers of cells based upon immunoreaction to different markers within the same field from three independent experiments. Data are expressed as the means ± SD.

### 2.5. Cell-Cycle Analysis

Cells (5 × 10^5^) were seeded in 60 mm plates, cultured in the appropriate medium, and incubated at 37°C with 5% CO_2_ for 48 hrs. Cell cultures were washed with PBS and detached with trypsin. For fixation and permeabilization, the cell suspension was washed once with PBS and resuspended in PBS containing 70% cold ethanol at 4°C for 1 hr. After two washes with PBS, the cells were treated at 37°C for 15 min with 40 *μ*g/mL RNase in PBS (final volume 100 *μ*L). Finally, propidium iodide (PI) staining solution (50 *μ*g/mL PI and 3.8 mmol/L sodium citrate in PBS) was added to the cells, which was followed by 3 hrs of incubation in the dark at 4°C. Cell-cycle analysis was performed with a FACSCalibur flow cytometer (Becton Dickinson), and PI fluorescence (designated as FL-2 height in the histogram plots) was measured at 630 nm.

### 2.6. Detection of Stored Glycogen

The stored glycogen was detected by the periodic acid-Schiff (PAS) staining system (Sigma-Aldrich) according to the manufacturer's instructions. Briefly, cells previously incubated with the appropriate medium in either the presence or absence of 0.75 mmol/L sodium butyrate for 3 days were fixed with formalin-ethanol fixative solution at room temperature for 1 min. After rinsing in slowly running tap water for 1 min, the slides were then oxidized with periodic acid solution for 5 min and followed by Schiff's reagent for 15 min. After rinsing with tap water for 5 min, slides were counterstained with hematoxylin solution.

### 2.7. Gene Expression Profiling

Triplicated cells (5 × 10^6^) were lysed in Trizol Reagent (Invitrogen) and purified with magnetic beads of Agencourt Ampure (APN 000132, Beckman Coulter) according to the manufacturer's instructions. A total of 500 ng RNA was used for a double-round of cDNA synthesis by GeneChip WT PLUS Reagent kit. Target 2nd-cycle single-stranded cDNA (ss-cDNA) were fragmented and biotin labeled by terminal deoxynucleotidyl transferase (Affymetrix, CA), and this was followed by microarray  hybridization to GeneChip Rat Gene 1.0 ST arrays (Affymetrix) according to the manufacturer's protocol on the Affymetrix Fluidics Station 450. Raw microarray data were scanned by Affymetrix GeneChip Command Console (AGCC) installed in GeneChip Scanner 3000 7 G. The data were analyzed with Robust Multichip Analysis (RMA) algorithm using default analysis settings and global scaling as normalization method by Partek Genomics Suite 6.6 Values presented was log2 RMA signal intensity. Normalized data were further analyzed using one-way ANOVA to screen out the differential expression gene. Then, the Database for Annotation, Visualization, and Integrated Discovery was used to determine pathways and processes of major biological significance and importance based on the Gene Ontology (GO) annotation function and Kyoto Encyclopedia of Genes and Genomes (KEGG) pathway function.

### 2.8. Statistical Analysis

Results were expressed as the mean ± SD. At least three independent determinations of each parameter were compared among the groups by one-way ANOVA tests using SPSS 11.5 statistics software. *p* < 0.05 was considered statistically significant.

## 3. Results

### 3.1. EGF Time-Dependently Reverses TGF-*β*1-Induced Myofibroblast Phenotype of Hepatic Oval Cells

**Figure 1:**
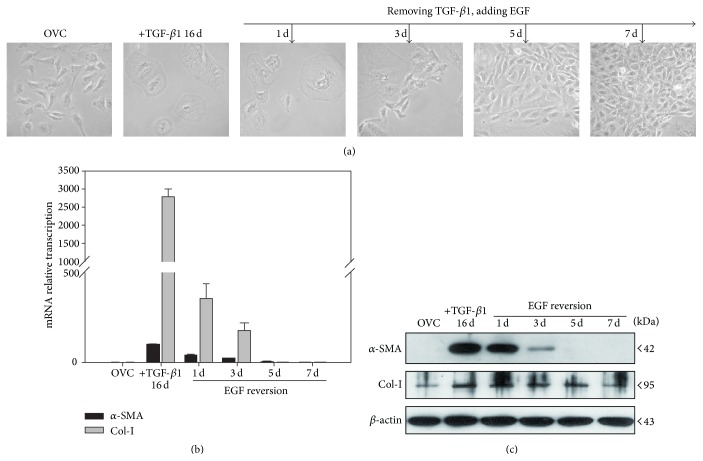
Seven-day EGF incubation allows for time-dependent reversal of the TGF-*β*1-induced myofibroblast phenotype in hepatic oval cells. (a) After 7-day EGF incubation, the morphology of 16-day TGF-*β*1-pretreated cells returned to an epithelial morphology similar to the control oval cells. ((b) and (c)) Incubation of 16-day TGF-*β*1-pretreated cells for 7 days with EGF reduced the mRNA transcription and protein expression of *α*-SMA and collagen I in a time-dependent manner.

Our previous results find that EGF is capable of both suppressing the initiation of TGF-*β*1-induced EMT and reversing cells that have previously undergone TGF-*β*1-induced EMT of hepatic oval cells [13]. In order to clarify the time-dependent loss of myofibroblast immunophenotype in the reversion process, 16-day TGF-*β*1 pretreated hepatic oval cells were incubated with EGF and observed at 0, 1, 3, 5, and 7 days, respectively. During the 7-day EGF-reversion process, the percentage of cells with epithelial morphology increased from 0% to 95% (Figure 1(a)). Compared to 16-day TGF-*β*1 treated cells, the mRNA transcription and protein expression of *α*-SMA and Col-I were reduced time-dependently approaching the levels found in control cells after 7-day EGF reversion (Figures 1(b) and 1(c)). These data suggest a time-dependent reversion process of EGF on TGF-*β*1-induced myofibroblast phenotype in hepatic oval cells.

### 3.2. Hepatic Oval Cells Retains Their Progenitor Immunophenotype in TGF-*β*1-Induced EMT and EGF-Induced Reversion Process

**Figure 2:**
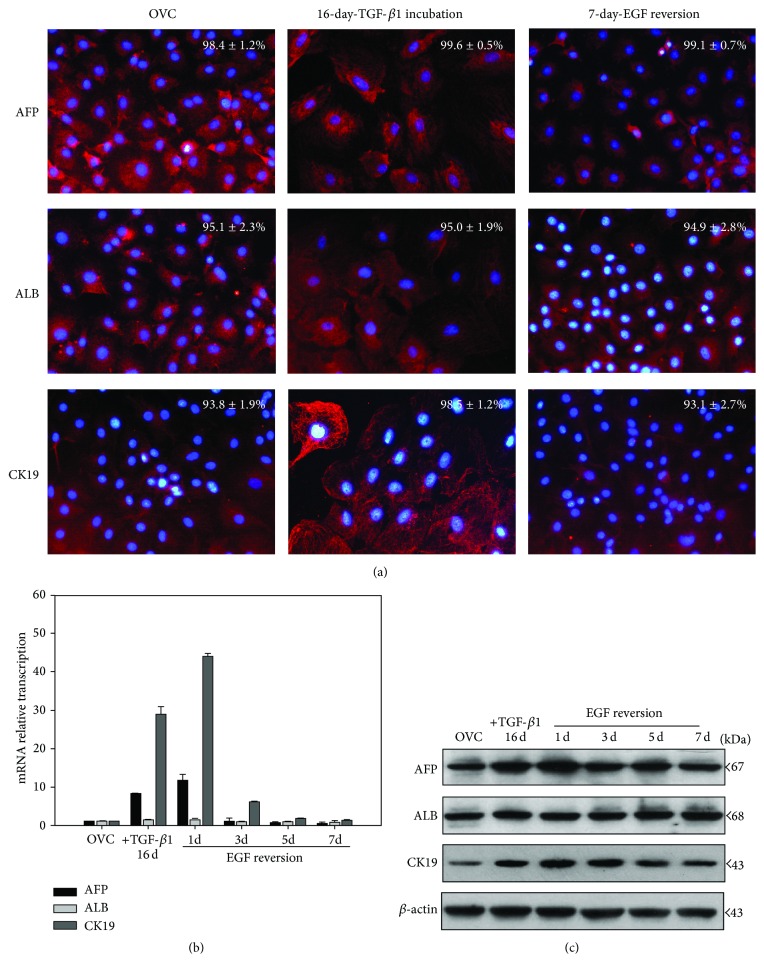
Hepatic oval cells maintained their progenitor immunophenotype in the EMT/MET process. (a) Hepatic oval cells expressed AFP, ALB, and CK19 after 16-day TGF-*β*1 incubation and 7-day EGF-reversion cells. (b) Real-time PCR analysis showed that 16-day TGF-*β*1 incubation increased AFP and CK19 transcription of hepatic oval cells, while EGF time-dependently reduced AFP and CK19 transcription to the level of control cells at 7 d. ALB transcription did not change during the EMT/MET process. (c) Western blot analysis also showed that TGF-*β*1 increase AFP and CK19 expression, whereas EGF downregulated AFP and CK19 expression in a time-dependent manner. ALB expression also did not change during EMT/MET process.

In order to clarify the time-dependent progenitor immunophenotype variation in the EMT/MET process, hepatic oval cells pretreated with TGF-*β*1 for 16 days were incubated with EGF for 0, 1, 3, 5, and 7 days, respectively. Immunofluorescence data showed that hepatic oval cells were positive for the progenitor cell markers AFP, ALB, and CK19 and retained these markers expression in 16-day TGF-*β*1 incubated cells and 7-day EGF-reversed cells (Figure 2(a)). The mRNA transcription analysis showed that hepatic oval cells maintained the mRNA transcription of ALB and increased mRNA transcription of AFP and CK19 after 16-day incubation with TGF-*β*1, and EGF also did not change ALB transcription but time-dependently downregulated AFP and CK19 transcription to the level of the control cells (Figure 2(b)). The protein expression analysis confirmed the sustained expression of ALB during the EMT/MET process, while TGF-*β*1 increased AFP and CK19 expression and EGF reverse this two genes expression to the level of control cells (Figure 2(c)).

### 3.3. EGF Restores the Proliferation Capacity, Which Could Be Inhibited by TGF-*β*1, in Hepatic Oval Cells

**Figure 3:**
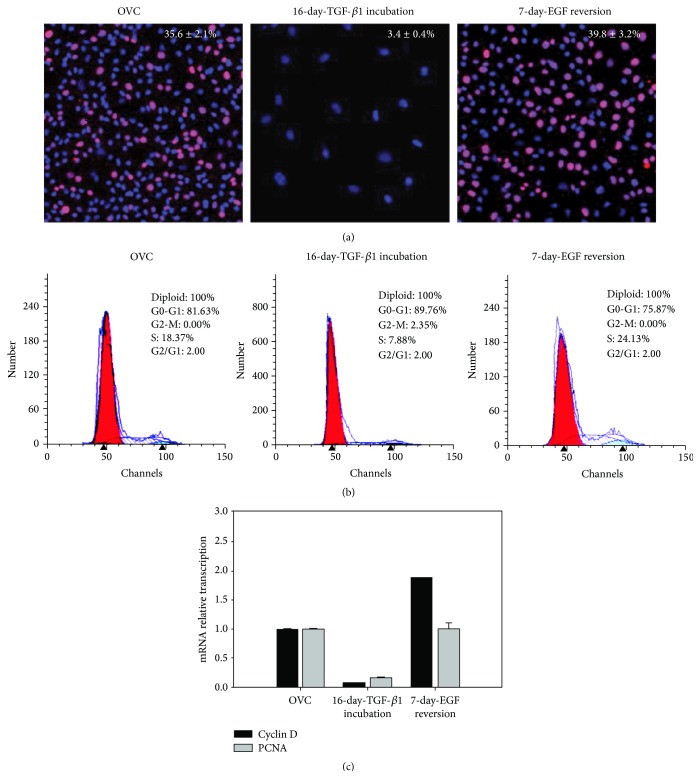
Seven-day EGF incubation restores the proliferation capacity of hepatic oval cells, which could be inhibited by TGF-*β*1. (a) Sixteen-day TGF-*β*1 incubation significantly reduced Ki-67 positive rate of hepatic oval cells; 7-day EGF incubation of 16-day TGF-*β*1-pretreated cells returned the Ki-67 positive rate to levels comparable to control cells. (b) Cell-cycle analysis by PI staining further demonstrated the rescue effects of 7-day EGF incubation on cell proliferation of 16-day TGF-*β*1-pretreated cells. (c) Real-time PCR analysis showed that 16-day TGF-*β*1 incubation reduced cyclin D and PCNA transcription, while 7-day EGF reversion could increase the mRNA transcription of cyclin D and PCNA to the level of control cells.

Previously, we find that TGF-*β*1 inhibits the proliferation capacity of hepatic oval cells [8], and whether EGF-induced reversion could rescue this capacity is necessary to be determined. TGF-*β*1 reduced the percentage of cells with positivity for Ki-67, a marker of proliferation from 35.6% of control oval cells to 3.4% after 16-day incubation, while 7-day EGF reversion recovered the number of Ki-67-positive cells to 39.8% of the 16-day TGF-*β*1-pretreated cells (Figure 3(a)). Similarly, the results of PI staining and cell-cycle analysis confirmed the findings of TGF-*β*1 inhibition of the proliferation of hepatic oval cells with the percentage of cells in active cell cycle being reduced from about 20% to 10%. Seven-day EGF reversion allowed approximately 25% of cells to be rescued and return to active mitosis (Figure 3(b)). Compared to the control cells, the mRNA transcription of cell proliferation related genes, cyclin D and PCNA, was inhibited in 16 d-TGF-*β*1 treated cells and returned to the control level after 7-day EGF-induced reversion (Figure 3(c)). These data indicate that EGF restores the proliferation capacity of the 16-day TGF-*β*1 treated hepatic oval cells.

### 3.4. EGF Retains the Differentiation Capacity, Which Could Be Impaired By TGF-*β*1, in Hepatic Oval Cells

**Figure 4:**
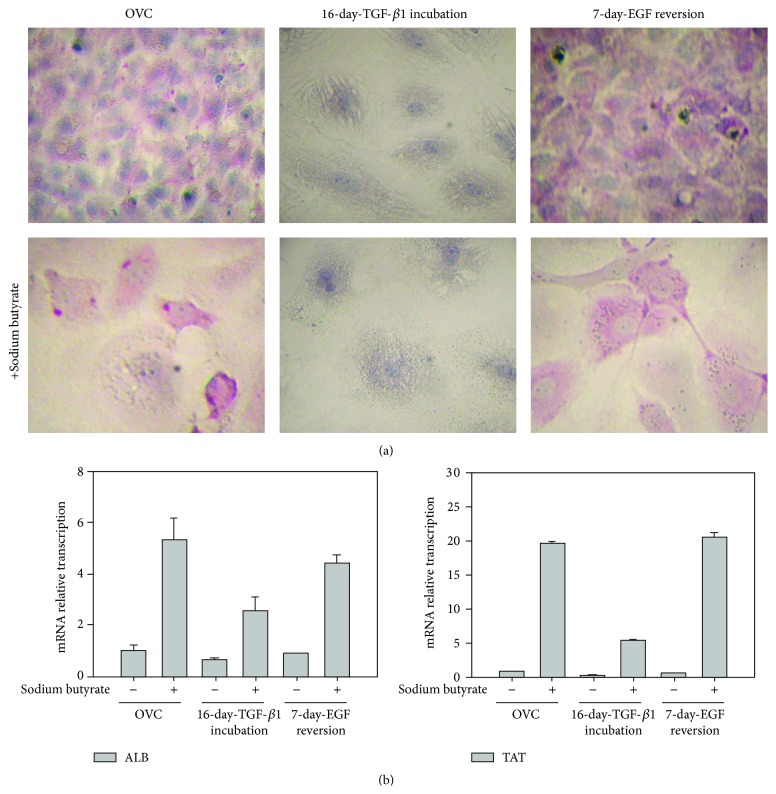
EGF retained the differentiation potential that was impaired by TGF-*β*1 in hepatic oval cells. (a) Hepatocyte differentiation, detected by PAS staining, could be induced by sodium butyrate in control oval cells and cells allowed to 7-day EGF recover from TGF-*β*1 exposure whereas hepatocyte differentiation could not be seen in cells after 16-day TGF-*β*1 incubation. (b) Sodium butyrate increased more ALB and TAT transcription in control oval cells and EGF-reversion cells than in 16-day TGF-*β*1 treated cells.

Besides proliferation, differentiation is another important function of hepatic progenitors. Hepatocyte differentiation was induced by adding sodium butyrate (0.75 mM) to the medium of control oval cells, 16-day TGF-*β*1-treated cells, and the 7-day EGF-reversion cells, respectively. Thereafter, PAS staining was utilized to observe the presence or absence of glycogen storage, as a measure for hepatocyte differentiation. Glycogen storage was observed in both the control and the reversion oval cells, especially after adding sodium butyrate (Figure 4(a)). However, no glycogen was detected in the 16-day TGF-*β*1-pretreated cells, either in the presence or absence of sodium butyrate (Figure 4(a)). Furthermore, sodium butyrate induced more ALB and TAT expression in control cells and 7-day EGF-reversion cells than in 16-day TGF-*β*1 treated cells (Figure 4(b)), suggesting that TGF-*β*1-induced EMT makes hepatic oval cells lose some hepatocyte-differentiation capacity.

### 3.5. Most of the Genes Induced by TGF-*β*1 Are Reversed by EGF to the Level of the Control Hepatic Oval Cells

**Figure 5:**
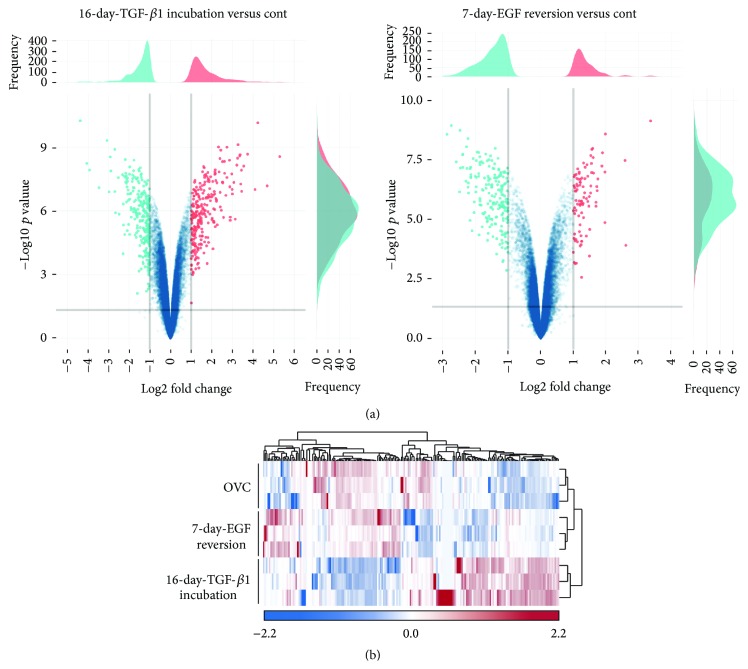
Differential gene expression of hepatic oval cells, 16-day TGF-*β*1 treated cells, and 7-day EGF-reversion cells. (a) Volcano plots contrast significance as the negative logarithm of the *p* value against log2 fold change between 16-day TGF-*β*1 treated versus control and 7-day EGF reversion versus control cells. (b) Hierarchical clustering analysis based on 200 genes significantly differentially expressed between hepatic oval cells, 16-day TGF-*β*1 treated oval cells, and 7-day EGF-reversion oval cells. Scaled expression values were shown for each group with light blue being the lowest and light red as the highest expression level.

To explore the extent of characteristic variation in hepatic oval cells experiencing EMT and MET, Affymetrix GeneChip microarray analysis was carried out on 16-day TGF-*β*1 treated cells and 7-day EGF-reversion cells, comparing them to untreated control oval cells. After raw data normalization and probe set summary, the expression values of 45,664 probe sets were analyzed for differential expression between 16-day TGF-*β*1 treated versus control and 7-day EGF treated versus control cells. This comparison revealed 256 genes with significant changes (−1.0 < log2 fold change > 1.0, i.e., cutoff values were set at more than 2.0-fold or less than 1/2-fold) in the transcript expression after 16-day TGF-*β*1 incubation, while there were only 91 genes with such significant changes if we compared 7-day EGF-reversion cells to control cells (Figure 5(a)). The log2 fold changes of 16-day TGF-*β*1 treated cells ranged from −5.0 to 6.0, while those of 7-day EGF-reversion cells ranged from −3.0 to 4.0, suggesting that most of the genes regulated by TGF-*β*1 are reversed by EGF (Figure 5(a)). A hierarchical clustering analysis based on 200 significantly differentially expressed genes showed two distinct groups among the control oval cells, 16-day TGF-*β*1 treated cells, and EGF-reversed cells. The control oval cells and EGF-reversed cells were clustered into one group, while 16-day TGF-*β*1 treated cells were clustered into the other group (Figure 5(b)), further confirming EGF reversing TGF-*β*1 pretreated cells similar to the control hepatic oval cells.

### 3.6. TGF-*β*1 Reduces Cell Cycle and Cytochrome P450 Metabolism Pathways, While EGF Increases MAPK and PI3K-Akt Pathways

**Figure 6:**
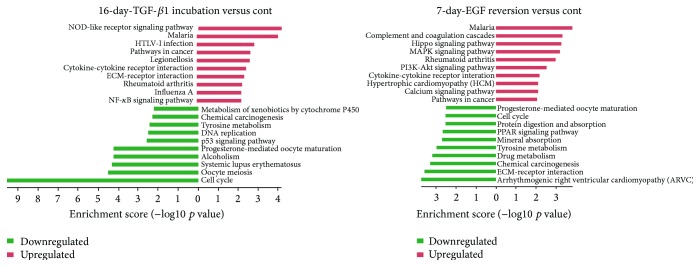
Top 10 upregulated (in red) and downregulated (in green) pathways in 16-day TGF-*β*1 treated cells and 7-day EGF-reversion cells compared to control hepatic oval cells.

To reveal the signal pathway involved in hepatic oval cells experiencing EMT and MET, pathway analysis was carried out by mapping genes onto the Kyoto Encyclopedia of Genes and Genomes (KEGG) database resources of the control oval cells, 16-day TGF-*β*1 treated cells, and 7-day EGF-reversion cells. The *p* value denotes the significance of the pathway, and the lower the *p* value is, the more significant change in the pathway is. The bar plot in Figure 6 showed the top ten enrichment score (−log10 *p* value) values of the significant enrichment pathways. Among the top 10 enrichment score values, 16-day TGF-*β*1 treatment greatly reduced the pathways of cell cycle, DNA replication, and metabolism of xenobiotics by cytochrome P450, which is consistent with our previous results where TGF-*β*1 impaired the proliferation and differentiation capacity but increased pathways involved in cancer, ECM-receptor interaction, and NF-*κ*B signaling if compared to control oval cells (Figure 6). Seven-day EGF reversion activated MAPK, PI3K-Akt, and calcium signaling pathway, yet it reduced the pathway of ECM-receptor interaction when compared to control cells (Figure 6). Cancer related pathways were still among the top 10 enrichment pathways in EGF-reversion cells (Figure 6), suggesting the maltransformation tendency after hepatic oval cells experiencing EMT and MET.

## 4. Discussion

The data presented here demonstrate that TGF-*β*1-induced transition maintains the progenitor immunophenotype of hepatic oval cells but abrogates their proliferation and differentiation characteristics. EGF-regulated reversion rescues most genes of TGF-*β*1-induced transition and recovered the proliferation and differentiation characteristics of hepatic oval cells through MAPK and PI3K-Akt signaling pathway. Although EMT/MET conversion activates cancer related signaling pathway, transient exposure to TGF-*β*1 or EGF does not induce malignant proliferation of hepatic oval cells.

Incubation with TGF-*β*1 does not abrogate the expression of hepatic progenitor cell markers but impaired the proliferation capacity and differentiation potential of hepatic oval cells. Our previous study shows that 48 hrs of TGF-*β*1 incubation induce a decrease in E-cadherin expression in hepatic oval cells but had no effect on the progenitor marker M2-isozyme of pyruvate kinase. In the current study, extending TGF-*β*1 incubation time to 16 days the cells retain their ALB expression and increased expression of AFP and CK19 and sustained TGF-*β*1 incubation abrogates proliferation ability and differentiation potential of hepatic progenitors with the impaired cell cycle, DNA replication, and metabolism of xenobiotics by cytochrome P450 signal pathway. Similar results are reported by Wu et al. [9], who show that long-term exposure to TGF-*β* (0.25 ng/mL for 18 weeks) leads to increased AFP expression in rat pluripotent liver progenitor-like WB-F344 cells with impaired liver progenitor cells' potential. So, the proliferation and differentiation function of hepatic progenitors are impaired in the microenvironment of liver fibrosis and cirrhosis, resulting in failing to regenerate the liver.

Previously, we report that EGF not only suppresses but also reverses TGF-*β*1-induced transition through phosphorylation of ERK1/2 and Akt [13]. In this study, we confirm our previous results by array data analysis that EGF activates MAPK and Akt signaling pathway during reversing TGF-*β*1 pretreated cells, resulting in recovering the proliferation and differentiation potential of hepatic progenitors which could be impaired by TGF-*β*1. It has been reported that PI3K signaling of HGF/MET is required for the survival of hepatic oval cells against TGF-*β*1 induced apoptosis [15], which indicates that, similar to HGF, EGF also serves as a counteract cytokine of TGF-*β*1.

Our data also reveals that both EMT and MET processes will activate signaling pathway for maltransformation of hepatic progenitors, which is consistent with the concept that epithelial cells dynamically switching between mesenchymal and epithelial phenotypic cellular states are involved in cancer metastasis [16]. But the proliferation capacity analysis in this study does not show any malignant proliferation signature of the TGF-*β*1-induced cells and EGF-regulated reversion cells because TGF-*β*1 suppresses the proliferation of hepatic oval cells and EGF reverses the proliferation capacity similar to that of the control oval cells. And we have shown that hepatic oval cells have no tumor initiation capacity even after long-term serial passages [14]. Data from Wu et al. [9] find that 18-week TGF-*β* exposure grants WB-F344 cells tumor initiating capacity, suggesting that maltransformation requires long-term and sustained activation of cancer related pathway. So, short-term or transient exposure to TGF-*β*1 or EGF will not cause maltransformation of hepatic oval cells.

In summary, EGF, the major cytokine responsive for liver regeneration, could reverse the characteristics that have been impaired by TGF-*β*1 in hepatic oval cells, serving as a protective cytokine to maintain the function of hepatic progenitors.

## Supplementary Material

This table showed the primer sequences used in this manuscript for real-time PCR analysis.
