# Supplementary material for: The Characteristics Variation of Hepatic Progenitors after TGF-β1-Induced Transition and EGF-Induced Reversion
Source: Stem Cells Int. 2016 Feb 3;2016:6304385. doi: 10.1155/2016/6304385 (PMC4756202; doi:10.1155/2016/6304385)
Supplement: Supplementary file 1 — This table showed the primer sequences used in this manuscript for real-time PCR analysis. [file 6304385.f1.pdf]

rvdTABLE S1. Primers Designed With Rat-Specific Genes for RT-PCR

Name	Accession no.	Primer sequence (5’-3’)	Product (bp)
AFP	NM_012493	GGAGAAGTGCTGCAAAGACC	120
		TTGTCCTTTCTTCCTCCTGG	
ALB	NM_134326	AGAACCAGGCCACTATCTC	110
		CAGATCGGCAGGAATGTTGT	
α-SMA	NM_031004.2	F: GTCCCAGACACCAGGGAGTGA	102
		R: TCGGATACTTCAGGGTCAGGA	
CK19	NM_199498	CAGCAGTATTGAAGTCCAGC	139
		TCAAGCAGGCTTCGGTAGGT	
Col-I	NM_053304.1	F: CATGTTCAGCTTTGTGGACCT	94
		R: GCAGCTGACTTCAGGGATGT	
GAPDH	NM_017008.4	F: CCTGCCAAGTATGATGACATCAAGA	75
		R: GTAGCCCAGGATGCCCTTTAGT	
